# Combined Histological and Proteomic Analysis Reveals Muscle Denervation in KMT5B-Related Neurodevelopmental Disorder: A Case Report

**DOI:** 10.3390/jcm14248636

**Published:** 2025-12-05

**Authors:** Ozge Aksel Kilicarslan, Andrea Gangfuß, Heike Kölbel, David Muhmann, Kiran Polavarapu, Rachel Thompson, Linda-Isabell Schmitt, Lola Lessard, Lei Chen, Astrid Eisenkölbl, Ulrike Schara-Schmidt, Andreas Hentschel, Hanns Lochmüller, Andreas Roos

**Affiliations:** 1 Children’s Hospital of Eastern Ontario Research Institute, Ottawa, ON K1H 8L1, Canada; oakselkilicarslan@cheo.on.ca (O.A.K.); kinnudreamz@gmail.com (K.P.); rae.thompson@gmail.com (R.T.); astrid.eisenkoelbl@kepleruniklinikum.at (A.E.); roos@andreas-roos.de (A.R.); 2 Department of Cellular and Molecular Medicine, University of Ottawa, Ottawa, ON K1H 8M5, Canada; 3 Department of Pediatric Neurology, Centre for Neuromuscular Disorders, Centre for Translational Neuro- and Behavioral Sciences, University Duisburg-Essen, 45147 Essen, Germany; andrea.gangfuss@uk-essen.de (A.G.); heike.koelbel@uk-essen.de (H.K.); david.muhmann@uk-essen.de (D.M.); leichen2023@gmail.com (L.C.); ulrike.schara-schmidt@uk-essen.de (U.S.-S.); 4 Department of Neurology, University Duisburg-Essen, 45147 Essen, Germany; linda-isabell.schmitt@uk-essen.de; 5 Service Troubles du Mouvement et Pathologies Neuromusculaires, Hôpital Neurologique, Groupement Hospitalier Est, Hospices Civils de Lyon, 69500 Bron, France; lola.lessard@chu-lyon.fr; 6 Institut NeuroMyoGène (INMG), Pathophysiology and Genetics of Neuron and Muscle (PGNM), CNRS UMR 5261-INSERM U1315, Université Claude Bernard Lyon 1, Faculté de Médecine, 69008 Lyon, France; 7 Department of Paediatrics and Adolescent Medicine, Johannes Kepler University Linz, Kepler University Hospital, 4020 Linz, Austria; 8 Leibniz-Institut für Analytische Wissenschaften, ISAS, e.V., 44139 Dortmund, Germany; andreas.hentschel@isas.de; 9 Brain and Mind Research Institute, University of Ottawa, Ottawa, ON K1N 8M5, Canada; 10 Division of Neurology, Department of Medicine, The Ottawa Hospital, Ottawa, ON K1H 8L6, Canada; 11 Centro Nacional de Análisis Genómico (CNAG), 08028 Barcelona, Spain

**Keywords:** KMT5B, muscle denervation in neurodevelopmental disorders, muscle proteomics, fast-twitch fiber predominance, hypotonia, case report

## Abstract

**Background**: Patients with neurodevelopmental and neuromuscular disorders often show overlapping clinical phenotypes. Pathogenic variants in *KMT5B*, a histone lysine methyltransferase, have been linked to neurodevelopmental disorders, yet their effects on human skeletal muscle remain unexplored. We report on a patient with *KMT5B*-linked disease who presented to a neuromuscular specialty clinic with significant involvement of skeletal muscle, where a multi-omics approach established the genetic diagnosis and revealed neuromuscular findings relevant for diagnosis, care and rehabilitation. **Methods**: Whole-exome sequencing was performed from blood and data was analyzed using the RD-Connect Genome Phenome Analysis Platform. Histological analysis and proteomic profiling were performed on muscle tissue. **Results**: Whole-exome sequencing revealed a pathogenic heterozygous variant (c.554_557del, p.Tyr185Cysfs*27) in *KMT5B*. Histological examination revealed fiber-type grouping, angular fibers, increased fast-twitch fiber proportion, and lipid droplet accumulation, indicative of muscle denervation. Proteomic profiling identified 77 dysregulated proteins, including upregulation of sarcomeric proteins, mitochondrial and glycolytic enzymes, acute-phase and complement factors, and extracellular matrix components, reflecting structural remodeling, metabolic adaptation, and inflammatory activation. These findings align with the role types observed in *Kmt5b* mouse models, supporting a role of *KMT5B* in neuromuscular function. **Conclusions**: We present the first combined histological and proteomic analysis of quadriceps muscle from a patient carrying a pathogenic *KMT5B* variant with a neuromuscular phenotype. The convergence of histological and proteomic alterations suggests that *KMT5B* haploinsufficiency may be associated with fiber-type shifts, denervation, and metabolic stress in human skeletal muscle. Understanding these processes provides mechanistic insight into motor deficits and informs targeted therapeutic strategies, including physiotherapeutic interventions, and early compensatory measures.

## 1. Introduction

KMT5B (Lysine-specific methyltransferase 5B) is a member of the SET-domain-containing family of proteins that function as histone lysine methyltransferases. It primarily catalyzes the methylation of histone H4 at lysine 20 (H4K20), a modification that is closely linked to chromatin compaction, gene silencing, and the regulation of DNA damage response pathways. Thus, KMT5B plays a crucial role in maintaining genomic stability, regulating cell cycle progression, and facilitating DNA repair [1,2]. Additionally, it has been implicated in the regulation of transcriptional repression and is thought to interact with a variety of co-factors that mediate its chromatin-modifying activities [3].

Heterozygous mutations in *KMT5B*, including missense, nonsense, and deletion variants, have been linked to a variety of phenotypes, ranging from developmental delay and autism spectrum disorders to more severe intellectual disability (MIM: 617788) [4]. These variants often disrupt the protein’s ability to properly regulate gene expression, leading to altered neuronal differentiation, synaptic function, and cognitive development [4,5]. The identification of these variants has underscored the critical role KMT5B plays in neuronal development and maintenance [4,6]. In addition, the role of *KMT5B* in myogenesis through regulation of the expression of target genes such as *EID3* has been described [7].

Sheppard et al. reported that hypotonia (22/43; 51%) and congenital heart defects (8/43; 18%) were also prominently associated with pathogenic variants in this gene in the largest *KMT5B* patient cohort (*n* = 43) to date, suggesting an expanded phenotype beyond autism spectrum and intellectual disability [4].

Motor deficits (motor developmental delay, joint hypermobility, joint laxity, hypotonia, poor suck and feeding difficulties) have been reported in patients with heterozygous *KMT5B* variants [8,9,10,11]. In a recent literature review assessing genotype-phenotype correlation in *KMT5B*- related syndrome, hypotonia was reported in 58% of patients [12]. However, these studies lack detailed clinical and pathological descriptions of the muscle phenotype, and this gene is not commonly recognized as a neuromuscular disease (NMD) gene. As a result, it may not be considered in the differential diagnosis of patients presenting with a neuromuscular phenotype. Kmt5b haploinsufficiency in mouse models indicates the role of KMT5B in neuromuscular function, including its effects on skeletal muscle mass, neuromuscular junction (NMJ) structure, and myofiber type, size, and distribution. Detailed phenotyping in the mouse revealed progressive muscle weakness and a reduction in muscle weight. Furthermore, *Kmt5b* haploinsufficiency was shown to increase NMJ fragmentation and alter myofiber composition, with an increase in fast-twitch fibers but a decrease in their size [6].

We present a patient attending a neuromuscular specialty clinic for a suspected neuromuscular/neurodevelopmental condition in whom *KMT5B*-related disease was established by multi-omics approaches. This study provides the first description of histological and biochemical findings from a quadriceps muscle biopsy in a patient carrying a pathogenic *KMT5B* variant and presenting with a neuromuscular phenotype. We demonstrate an increased proportion of fast-twitch fibers, accompanied by frequent fiber-type grouping and the presence of angular fibers, features consistent with denervation, similar to observations made in the mouse model.

## 2. Materials and Methods

### 2.1. Clinical Data Collection

The study was conducted in accordance with the Declaration of Helsinki, and approved by the Ethics Committee of University Medical Centre, Essen, Germany (19-9011-BO) on 30 April 2020. Informed consent was obtained from all participants involved in the study. Clinical data were collected from the medical records.

### 2.2. Whole-Exome Sequencing (WES) and Variant Interpretation

DNA was extracted from the blood sample of the patient. WES was performed using the Twist HumanRefSeq Panel to identify the underlying genetic cause. Raw data in FASTQ format was uploaded to the RD-Connect Genome Phenome Analysis Platform (GPAP) (https://platform.rd-connect.eu (accessed on 01 April 2025)) for processing and variant calling, and the GPAP platform was used to perform bioinformatic analysis and variant prioritization. Variants were filtered based on protein impact (e.g., stop gained, missense, frameshift) and variant population frequency (<0.01 MAF in gnomAD v4.1.0). The putative effect of shortlisted variants was assessed using multiple in silico prediction tools including Combined Annotation Dependent Depletion (CADD), polymorphism phenotyping v2 (PolyPhen-2), Sorting Intolerant from Tolerant (SIFT), Rare Exome Variant Ensemble Learner (REVEL) and AlphaMissense (all accessed on 01 April 2025 via API access from the RD-Connect GPAP, https://platform.rd-connect.eu).

### 2.3. Microscopic Studies

All stains were performed on 8 μm cryo-microtome sections according to standard procedures. Immunohistochemical staining procedures were carried out as described previously [13]. Antibody catalog numbers and dilutions are listed in Supplementary Table S1. Biochemical measurement of the respiratory chain enzymes was performed at a German reference laboratory, the Friedrich-Baur-Institute in Munich according to the methodology described previously [14].

### 2.4. Proteomic Profiling

Unbiased proteomic profiling was carried out in a data-independent-acquisition (DIA) mode on whole protein extracts of quadriceps muscle biopsies derived from our index and four age- and sex-matching controls using methodology described previously [15]. Patient-derived samples were analyzed to technical triplicates to enable statistical analysis.

For the analysis of the samples acquired with nano-LC-MS/MS in DIA mode, the data was entered into the Spectronaut software (v.14.10.201222, Biognosys, Schlieren, Switzerland) and analyzed with a direct DIA-based search. As library, the human proteome data was selected from UniProt (https://www.uniprot.org (accessed on 15 September 2025)) containing 20,404 entries. Search and extraction settings were kept as standard (BGS Factory settings). Normalization was done by the software, using global normalization based on the median. The false discovery rate (FDR) was set to 1% for peptide-spectrum matches (PSMs), peptides, and proteins. There was no batch correction done, as the samples (patient and control samples) were analyzed in one single batch.

For reliable label-free quantification, only proteins identified with ≥2 unique peptides were considered for further analysis. Subsequently, the average normalized abundances (determined using Spectronaut, v.14.10.201222, Biognosys, Schlieren, Switzerland) were calculated for each protein and used to determine the ratio between the leukocytes from the patients and the corresponding controls. Finally, a Student’s *t*-test with *p*-values was calculated in MS Excel. Only proteins with a *p*-value of ≤0.05 and an abundance ratio of ≥2 or ≥0.5 (up-regulated as well as down-regulated) were considered as finally regulated.

## 3. Results

### 3.1. Case Presentation

The patient was a Caucasian female born at term via spontaneous vaginal delivery to non-consanguineous parents after a pregnancy notable for reduced fetal movements. Birth weight was 3900 g and length was 49 cm. The neonatal period was unremarkable. Both parents were reported healthy. The patient has a healthy half-sister, a brother with attention deficit hyperactivity disorder (ADHD), a maternal grandmother with multiple sclerosis, and a paternal grandfather with Parkinson’s disease.

Psychomotor developmental delay was evident from infancy, with poor suck, hypotonia, and late motor and language milestones (rolling at 12 months, walking at 2.5 years, delayed first words). A congenital atrial septal defect was diagnosed at 2 years of age and surgically corrected soon after. From early childhood, the patient’s parents reported episodes of “sudden developmental regression” characterized by fatigue, hypersomnia, and temporary stiffening of her left leg, which led to falls. This was followed by further developmental improvements, although she never reached a level of development appropriate for her age. Over time, she showed reduced muscle strength and endurance with progressive fatigability. Between the ages of 3 and 7 the patient attended a special education kindergarten for children with developmental disabilities and subsequently enrolled in a school for physically disabled children.

At the age of 14, she was first referred to our outpatient clinic for evaluation of an underlying muscle disease. At that time, neuromuscular symptoms were observed including walking difficulty (maximum walking distance of 500 m), exertional muscle cramps, and frequent choking episodes. In daily life she required assistive devices including a wheelchair for longer distances, lower leg orthoses, and a mechanical lift for bathing. Additionally, she reported joint and bone pain and exertional headaches. She also had a mild intellectual disability.

Anthropometric measurements were within the normal range. Head circumference, weight and height were at 50th, 50th and between 25th and 50th percentile respectively. No facial dysmorphism except strabismus was noted. Physical examination revealed genua valga, mild scapulae alatae, bilateral pes planovalgus, and a waddling gait. Neurological assessment demonstrated mild distal-predominant hypotonia, bilateral weakness on single-leg stance, mild postural tremor, distal hyperlaxity/hyperextensibility, mild bilateral dysdiadochokinesia, and subtle right-sided divergent strabismus.

### 3.2. Diagnostic Workup

Neuroimaging at ages 4 and 9 years showed mild asymmetry of the lateral ventricles; MRI at age 14 was normal. EEG at ages 11 and 14 revealed generalized abnormalities with hyperventilation-induced slowing but no epileptiform activity. Visual evoked potentials at age 13 showed prolonged right-sided P100 latency, consistent with right visual pathway dysfunction. Nerve conduction studies (N. peronaeus, N. suralis, N. medianus) showed no pathology. EMG was not performed. Chromosomal analysis at age 8 demonstrated a normal female karyotype. Orthopedic evaluation at age 10 showed a left-convex lumbar scoliosis (Cobb angle 7°). Exercise testing at age 14 revealed markedly elevated post-exercise lactate (57.2 vs. 10.4 mg/dL baseline), suggesting impaired metabolic or mitochondrial function. At the age of 16, a quadriceps biopsy was performed for a suspected diagnosis of mitochondriopathy.

### 3.3. Whole-Exome Sequencing

WES revealed a heterozygous deletion of four nucleotides (hg38 chr11:68173899 CAAAT>C, c.554_557del) in *KMT5B* (NM_017635.5) causing a frameshift starting at Tyrosine (Tyr) 185 and resulting in a premature stop codon 27 residues later (p.Tyr185Cysfs*27). The quality control parameters included a genotype quality score of 99 and a sequencing depth of 46. WES data is available to registered users of the RD-Connect GPAP. Population minor allele frequency (MAF) for this variant was 0.0000006242 in gnomAD V4.1. (1 heterozygote and no homozygotes). The internal frequency of this variant in the RD-Connect GPAP was 0.00004 with no other patient with the same variant. This variant was reported as a de novo pathogenic variant in a patient with autism spectrum disorder (ASD) [16] and is present in the ClinVar database. We used Franklin variant interpretation platform (https://franklin.genoox.com/clinical-db/home (accessed on 01 April 2025)) for determining pathogenicity using the American College of Medical Genetics and Genomics (ACMG) criteria. It is classified as pathogenic as per the ACMG criteria PVS1, PM2, and PS4 [17]. Segregation analysis was not performed as parents were not able to give samples. No other significant variants, CNVs or structural variants in the coding regions or splice junctions were found.

### 3.4. Microscopic Findings

**Figure 1:**
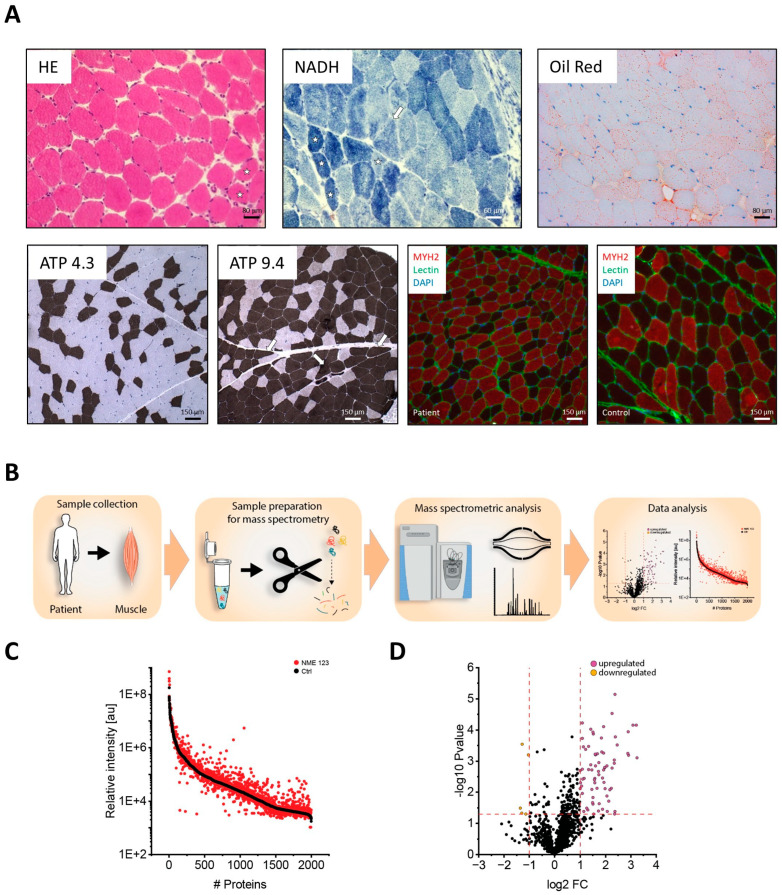
Histopathological and Proteomic Signatures in Patient Muscle Biopsy: (**A**) Histological analysis of the muscle biopsy revealed fiber caliber variability (white stars in HE stain), presence of angular fibers (white arrows in NADH and ATPase stains) and marked sarcoplasmic increase of lipid droplets within fibers (Oil Red O stain). ATPase stains (pH 4.3 and 9.4) demonstrated a predominance of fast-twitch fibers. MYH2 stains showed a change in the distribution of fiber types and decrease in size. (**B**) Schematic overview of the proteomic profiling workflow applied to the patient’s muscle biopsy. (**C**) Protein abundance plot showing the dynamic range of all proteins identified in muscle cell extracts using liquid chromatography–tandem mass spectrometry (LC-MS/MS). Quantification was based on the three most abundant peptides per protein, enabling intra-experimental comparison. Control proteins (black) are ordered by decreasing abundance; patient proteins (red) are plotted in the same order to allow direct comparison. The identified proteins span a dynamic range of eight orders of magnitude. (**D**) Volcano plot highlighting proteins with statistically significant increases (purple) and decreases (yellow) in abundance (proteins in black showed no statistically significant change in abundance).

Histological analysis of the muscle biopsy (Figure 1A) revealed fiber caliber variability (white stars in HE stain), presence of angular fibers (white arrows in NADH and ATPase stains) and a marked accumulation of lipid droplets within fibers (Oil Red O stain). ATPase staining at pH 4.3 and 9.4 revealed a predominance of fast-twitch fibers, which stained dark at pH 9.4 and light at pH 4.3. MYH2 staining further demonstrated smaller fiber groupings and fast-twitch predominance, corroborating the ATPase findings. While not conclusive in the absence of corroboratory evidence from EMG testing (not performed), taken together, these findings are nevertheless suggestive of chronic, skeletal muscle denervation. Biochemical measurement of the respiratory chain enzymes (complexes I to IV) showed normal activity. Numerical values and normal ranges are listed in Supplementary Table S2.

### 3.5. Proteomic Findings

Proteomic profiling of quadriceps muscle from the patient (Figure 1B) enabled the robust identification of 2061 proteins (covering a dynamic range of eight orders of magnitude) in controls and our patient (Figure 1C) and revealed a broad dysregulation of a total of 77 proteins (72 are increased and 5 are decreased; Figure 1D). Increased abundance of sarcomeric proteins, including ACTA1, TNNI2, and MYL2 was also identified and indicated remodeling of the contractile apparatus, consistent with the histological finding of a shift toward fast-twitch fibers. Multiple mitochondrial and glycolytic enzymes, including NDUFB3, NDUFB8, LDHB, PKLR, and PRDX2, were upregulated, suggesting metabolic stress with concomitant adaptations in energy production and redox balance. Elevations in SLC2A1 and BPGM further supported altered glucose handling. A striking enrichment of acute-phase proteins (A2M, SERPINA1/3, HP, ORM2), complement factors (C3, C4BPA, CFB, C1QC), and inflammatory mediators (S100A8, S100A9) pointed to local inflammatory processes. In parallel, proteins associated with extracellular matrix remodeling and vascular interactions (VTN, ITIH1/2/4, LRG1) were also increased. Together, these findings highlight a complex proteomic signature encompassing structural remodeling, metabolic reprogramming, and inflammatory activation in the skeletal muscle of the patient. The mass spectrometry proteomics data have been deposited to the ProteomeXchange Consortium via the PRIDE partner repository with the dataset identifier PXD071037.

## 4. Discussion

*KMT5B* encodes a lysine methyltransferase responsible for catalyzing methylation of histone H4 at lysine 20 (H4K20me2), a key mark involved in chromatin organization, DNA replication, and genome stability. Pathogenic dominant variants in *KMT5B* have been associated with neurodevelopmental disorders, intellectual disability, autism spectrum disorder, and, in some cases, overgrowth phenotypes [11]. While hypotonia has been described earlier, previous studies were lacking in description of neuromuscular involvement in these patients either clinically or pathologically. Our study reports a clinical and pathological pattern of neuromuscular involvement in addition to the neurodevelopmental phenotype in a patient with a heterozygous *KMT5B* truncating variant.

Phenotyping of a gene trap mouse model of Kmt5b showed muscle cell vulnerability including decrease in skeletal muscle mass, changes in neuromuscular junction (NMJ) structure, and altered myofiber type, size, and distribution (predominance of fast twitch fibers) [6]. These myopathological findings may indicate altered muscle innervation or denervation. Muscle denervation is compatible with the known role of KMT5B in neuronal processes. To date, no studies on human muscle derived from *KMT5B* patients have been described. Here, we provide the first combined morphological and biochemical study on a quadriceps biopsy collected for diagnostic purposes from a patient presenting with neuromuscular symptoms prior to the molecular genetic diagnosis of a *KMT5B*-related neurodevelopmental disorder.

Histological characterization revealed changes in fiber caliber, predominance of fast-twitch fibers and presence of angular fibers. In addition, an increase in lipid droplets in muscle fibers was observed. Lipid accumulation in muscle fibers is known to be associated with muscle denervation and atrophy in relation to myosteatosis [18] and has recently also been described in the context of *SLC18A3*-related congenital myasthenic syndrome, a NMJ disorder [19]. The proteomic alterations observed in muscle of our patient provide mechanistic insight into the muscle pathology associated with *KMT5B* haploinsufficiency. The upregulation of contractile proteins and the preferential increase in fast-twitch fiber markers align with histological evidence of fiber-type regrouping and angular fibers, suggesting that altered fiber identity and denervation may contribute to the phenotype. Elevated mitochondrial and glycolytic enzymes indicate metabolic adaptations, likely reflecting a compensatory response to impaired neuromuscular function and increased energetic demand. The strong representation of acute-phase, complement, and inflammatory proteins further suggests that immune activation may be a secondary feature of the disease process, possibly triggered by cycles of denervation and reinnervation. The enrichment of extracellular matrix and secreted proteins supports the view that remodeling of the muscle microenvironment accompanies these processes. The convergence of metabolic, structural, and immune signatures strongly indicates a genuine disease-associated remodeling program. Taken together, these findings suggest that KMT5B dysfunction may lead to a multifaceted muscle pathology characterized by fiber-type shifts, metabolic stress, and inflammatory remodeling, features that may represent key biomarkers for future translational studies and potential therapeutic targets.

We suggest that *KMT5B*-related disease caused by heterozygous truncating variants extends beyond the previously described neurodevelopmental disorder to cause an additional neuromuscular pathophenotype. This is evidenced not only by the results of detailed phenotyping of a *Kmt5b* mouse model by others [6] but also by the clinical findings in our patient, who first presented to specialty services at a neuromuscular clinic with distal predominant hypotonia, muscle weakness, fatigability and hyperextensible distal joints. While further studies on *KMT5B* patients are needed to define the neuromuscular phenotype more precisely, these findings show that it is important to consider *KMT5B* in the differential diagnosis of patients presenting to a neuromuscular clinic with a complex neurodevelopmental plus neuromuscular phenotype.

For disorders with heterogeneous presentations such as neurodevelopmental conditions accompanied by neuromuscular symptoms, patients may benefit from evaluation by neuromuscular specialists and comprehensive diagnostic work-up. Conversely, in patients initially seen for neuromuscular disorders, it is equally important to consider underlying neurodevelopmental conditions that may contribute to the clinical picture. This case highlights the importance of considering genes not traditionally associated with neuromuscular disease, underscoring the need to broaden differential diagnoses in patients presenting with neuromuscular features and the importance of multi-omics approaches. For clinical practice, a confirmed genetic diagnosis may allow families to access early social support and genetic counseling for family planning [20], while knowledge of neuromuscular involvement will also enable these patients to receive appropriate multidisciplinary clinical care including the selection of appropriate medications and physiotherapeutic or rehabilitative methods. Greater recognition of the potential neuromuscular features when patients present to neurodevelopmental specialists may prompt more holistic care, as tailoring physiotherapy and rehabilitation interventions in patients with KMT5B-related disease to the neuromuscular impairments, such as muscle weakness and contractures, is essential for optimizing functional outcomes and preventing adverse effects from inappropriate therapeutic approaches.

## Data Availability

Genomic data are available to registered users of the RD-Connect GPAP. Other data presented in this study are available on request from the corresponding author due to privacy, legal or ethical reasons.

## Supplementary Materials

The following supporting information can be downloaded at: https://www.mdpi.com/article/10.3390/jcm14248636/s1, Table S1: Antibody Catalog Numbers. Table S2: Numeric values with reference ranges of the respiratory chain enzyme assays.

## Abbreviations

The following abbreviations are used in this manuscript:

A2M	Alpha-2-macroglobulin
ACTA1	Actin alpha 1
ADHD	Attention deficit hyperactivity disorder
APOA1/2/4	Apolipoprotein A1/2/4
APOB	Apolipoprotein B
APOE	Apolipoprotein E
ASD	Autism spectrum disorder
BPGM	Bisphosphoglycerate mutase
C1QC	Complement C1q C Chain
C3	Complement Component 3
C4BPA	Complement Component 4 Binding Protein Alpha
CADD	Combined Annotation Dependent Depletion
CFB	Complement factor B
EEG	Electroencephalogram
EID3	EP300 Interacting Inhibitor of Differentiation 3
gnomAD	The Genome Aggregation Database
GPAP	RD-Connect Genome Phenome Analysis Platform
H4K20	Histone H4 at lysine 20
HBA1	Hemoglobin subunit alpha 1
HBB	Hemoglobin subunit beta
HBD	Hemoglobin subunit delta
HBG1	Hemoglobin Subunit Gamma 1
HP	Haptoglobin
ITIH1/2/4	Inter-alpha-trypsin inhibitor, heavy chain 1/2/4
KMT5B	Lysine-specific methyltransferase 5B
LDHB	Lactate Dehydrogenase B
LRG1	Leucine-rich alpha-2-glycoprotein 1
MAF	Minor Allele frequency
MRI	Magnetic resonance imaging
MYL2	Myosin light chain-2
NDUFB3	NADH: Ubiquinone Oxidoreductase Subunit B3
NDUFB8	NADH: Ubiquinone Oxidoreductase Subunit B8
NMJ	Neuromuscular junction
ORM2	Orosomucoid 2
PKLR	Pyruvate Kinase L/R
PolyPhen-2	Polymorphism phenotyping v2
PRDX2	Peroxiredoxin 2
REVEL	Rare Exome Variant Ensemble Learner
S100A8	S100 calcium-binding protein A8
S100A9	S100 calcium-binding protein A9
SERPINA1/3	Serpin family A member 1/3
SIFT	Sorting Intolerant from Tolerant
SLC2A1	Solute carrier family 2, member 1
TNNI2	Troponin I2
VTN	Vitronectin

## References

[1] Bromberg K.D., Mitchell T.R.H., Upadhyay A.K., Jakob C.G., Jhala M.A., Comess K.M., Lasko L.M., Li C., Tuzon C.T., Dai Y. (2017). The SUV4-20 inhibitor A-196 verifies a role for epigenetics in genomic integrity. Nat. Chem. Biol..

[2] Wu H., Siarheyeva A., Zeng H., Lam R., Dong A., Wu X.-H., Li Y., Schapira M., Vedadi M., Min J. (2013). Crystal structures of the human histone H4K20 methyltransferases SUV420H1 and SUV420H2. FEBS Lett..

[3] Wang Z.-J., Rein B., Zhong P., Williams J., Cao Q., Yang F., Zhang F., Ma K., Yan Z. (2021). Autism risk gene KMT5B deficiency in prefrontal cortex induces synaptic dysfunction and social deficits via alterations of DNA repair and gene transcription. Neuropsychopharmacology.

[4] Sheppard S.E., Bryant L., Wickramasekara R.N., Vaccaro C., Robertson B., Hallgren J., Hulen J., Watson C.J., Faundes V., Duffourd Y. (2023). Mechanism of KMT5B haploinsufficiency in neurodevelopment in humans and mice. Sci. Adv..

[5] Chen G., Han L., Tan S., Jia X., Wu H., Quan Y., Zhang Q., Yu B., Hu Z., Xia K. (2022). Loss-of-function of KMT5B leads to neurodevelopmental disorder and impairs neuronal development and neurogenesis. J. Genet. Genom..

[6] Hulen J., Kenny D., Black R., Hallgren J., Hammond K.G., Bredahl E.C., Wickramasekara R.N., Abel P.W., Stessman H.A.F. (2022). KMT5B is required for early motor development. Front. Genet..

[7] Neguembor M.V., Xynos A., Onorati M.C., Caccia R., Bortolanza S., Godio C., Pistoni M., Corona D.F., Schotta G., Gabellini D. (2013). FSHD muscular dystrophy region gene 1 binds Suv4-20h1 histone methyltransferase and impairs myogenesis. J. Mol. Cell Biol..

[8] Wickramasekara R.N., Stessman H.A.F. (2019). Histone 4 Lysine 20 Methylation: A Case for Neurodevelopmental Disease. Biology.

[9] Faundes V., Newman W.G., Bernardini L., Canham N., Clayton-Smith J., Dallapiccola B., Davies S.J., Demos M.K., Goldman A., Gill H. (2018). Histone Lysine Methylases and Demethylases in the Landscape of Human Developmental Disorders. Am. J. Hum. Genet..

[10] Trinh J., Kandaswamy K.K., Werber M., Weiss M.E.R., Oprea G., Kishore S., Lohmann K., Rolfs A. (2019). Novel pathogenic variants and multiple molecular diagnoses in neurodevelopmental disorders. J. Neurodev. Disord..

[11] Eliyahu A., Barel O., Greenbaum L., Zaks Hoffer G., Goldberg Y., Raas-Rothschild A., Singer A., Bar-Joseph I., Kunik V., Javasky E. (2022). Refining the Phenotypic Spectrum of KMT5B-Associated Developmental Delay. Front. Pediatr..

[12] Politano D., Borgatti R., Borgonovi G., Cistaro A., Danesino C., Fania P., Garghetti G., Guala A., Orlando I., Schiera I.G. (2025). Bridging Genotype to Phenotype in KMT5B-Related Syndrome: Evidence from RNA-Seq, 18FDG-PET, Clinical Deep Phenotyping in Two New Cases, and a Literature Review. Genes.

[13] Dubowitz V., Sewry C.A., Oldfors A., Lane R.J.M. (2020). Muscle Biopsy: A Practical Approach: Expert Consult.

[14] Fischer J.C., Ruitenbeek W., Gabreëls F.J.M., Janssen A.J.M., Renier W.O., Sengers R.C.A., Stadhouders A.M., Ter Laak H.J., Trijbels J.M.F., Veerkamp J.H. (1986). A mitochondrial encephalomyopathy: The first case with an established defect at the level of coenzyme Q. Eur. J. Pediatr..

[15] Pauper M., Hentschel A., Tiburcy M., Beltran S., Ruck T., Schara-Schmidt U., Roos A. (2025). Proteomic Profiling Towards a Better Understanding of Genetic Based Muscular Diseases: The Current Picture and a Look to the Future. Biomolecules.

[16] Satterstrom F.K., Kosmicki J.A., Wang J., Breen M.S., De Rubeis S., An J.-Y., Peng M., Collins R., Grove J., Klei L. (2020). Large-Scale Exome Sequencing Study Implicates Both Developmental and Functional Changes in the Neurobiology of Autism. Cell.

[17] Richards S., Aziz N., Bale S., Bick D., Das S., Gastier-Foster J., Grody W.W., Hegde M., Lyon E., Spector E. (2015). Standards and guidelines for the interpretation of sequence variants: A joint consensus recommendation of the American College of Medical Genetics and Genomics and the Association for Molecular Pathology. Genet. Med..

[18] Gumucio J.P., Qasawa A.H., Ferrara P.J., Malik A.N., Funai K., Mcdonagh B., Mendias C.L. (2019). Reduced mitochondrial lipid oxidation leads to fat accumulation in myosteatosis. FASEB J..

[19] Della Marina A., Arlt A., Schara-Schmidt U., Depienne C., Gangfuß A., Kölbel H., Sickmann A., Freier E., Kohlschmidt N., Hentschel A. (2021). Phenotypical and Myopathological Consequences of Compound Heterozygous Missense and Nonsense Variants in SLC18A3. Cells.

[20] Fraiman Y.S., Wojcik M.H. (2021). The influence of social determinants of health on the genetic diagnostic odyssey: Who remains undiagnosed, why, and to what effect?. Pediatr. Res..

