# Supplementary material for: Combined Histological and Proteomic Analysis Reveals Muscle Denervation in KMT5B-Related Neurodevelopmental Disorder: A Case Report
Source: J Clin Med. 2025 Dec 5;14(24):8636. doi: 10.3390/jcm14248636 (PMC12733465; doi:10.3390/jcm14248636)
Supplement: Supplementary file 1 [file jcm-14-08636-s001.zip › jcm-3970880-supplementary.pdf]

Supplementary Material for Aksel Kilicarslan O et al: Combined Histological and Proteomic Analysis Reveals Muscle Denervation in KMT5B-Related Neurodevelopmental Disorder: A Case Report

Table S1: Antibody Catalog Numbers

Antibody	Host	Dilution	Best.-NR.	Firma
MYH2	Rabit	1:200	GTX100712	GeneTex

Table S2: Numeric values with reference ranges of the respiratory chain enzyme assays

Name	Activity	Normal range	Activity	Normal range
	Based on grams of non-collagen protein		Based on Citratsynthase	
Complex I	33.8 U/gNCP	15.8 – 42.8	0.43	0.17 – 0.56
Complex II / III	19.8 U/gNCP	6.0 – 25.0	0.25	0.08 - 0.45
Complex IV	174.6 U/gNCP	112 - 351	2.21	(1.1 – 5.0)
Citratsynthase (CS)	79.0 U/gNCP	45 - 100		
Non-collagen Protein (NCP)	7.0 g/l			
